# An Unusual Cause of Biliary Peritonitis on the Background of Acute Pancreatitis: A Case Report

**DOI:** 10.1055/s-0042-1756284

**Published:** 2022-09-02

**Authors:** Dimitrios Symeonidis, Efrosyni Bompou, Athina A. Samara, Labrini Kissa, Konstantinos Tepetes

**Affiliations:** 1 Department of Surgery, University Hospital of Larissa, Mezourlo, Larissa, Greece

**Keywords:** acute pancreatitis, biliary peritonitis, local complications, necrotic collections

## Abstract

**Introduction** Acute pancreatitis can cause a wide variety of local complications, sometimes pretty unusual. In the present report, we present a rather unusual cause of biliary peritonitis on the background of acute pancreatitis.

**Case Presentation** A 41-year-old female patient with biliary acute pancreatitis and concomitant choledocholithiasis required an urgent laparotomy due to signs of sepsis and peritoneal irritation after a trial of conservative management. During laparotomy, the diagnosis of biliary peritonitis was established. Surprisingly, a residual gallstone obstructing the common bile duct at the level of the ampulla was causing bile to reflux, through the common channel, into the main pancreatic duct and subsequently into a partially ruptured acute pancreatic necrotic collection.

**Conclusion** Dealing with the unexpected is a constant challenge for the surgical team dealing with acute pancreatitis patients. Although deferring surgical intervention during the course of acute pancreatitis, as much as possible, is the ideal strategy, this is not always possible. Deciding the treatment strategy based on the patients' clinical condition represents the most appropriate approach.

Acute pancreatitis (AP) is an inflammatory condition of the pancreas most commonly caused by bile stones or excessive use of alcohol. 1 One of the most frequent local complications of AP is the development of pancreatic or peripancreatic fluid collections. The revised Atlanta classification in 2012 has categorized these collections into four certain categories. More specifically, AP-associated collections involve acute peripancreatic fluid collections (APFCs), acute necrotic collections (ANCs), pancreatic pseudocysts (PPs), and walled-off necrosis (WONs). 1 2

APFCs are characterized by the absence of solid components. They typically arise in patients with edematous AP during the first 4 weeks after the onset of an episode of AP. Inflammation or even rupture of one or more small peripheral pancreatic side duct branches represents the possible causes of this type of collections. APFCs are usually seen in close proximity to the pancreas and have no identifiable wall. 1 A PP, on the other hand, is a well-defined collection with prominent wall, seen more than 4 weeks after the onset of interstitial edematous pancreatitis. By definition, there is no associated necrotic tissue in this collection. PPs may arise from the rupture of the main pancreatic duct or one side branch. However, as is the case with APFCs, not all PPs have an established communication with the pancreatic ductal system. 1

ANCs occur as a result of necrotizing AP. These collections contain both liquid and necrotic components. Within the first week of the disease, it may be difficult to differentiate APFCs from ANCs. 3 A peripancreatic fluid collection that is associated with an imaging documented pancreatic parenchymal necrosis, as well, is the diagnostic criterion of ANC. Finally, WON consists of necrotic tissue well contained within a matured wall of reactive tissue. Usually, this maturation occurs after 4 weeks after the onset of necrotizing AP. Both ANCs and WONs can become infected. The presence of infection can be suspected by the patient's clinical course or by the presence of gas within the collection as seen on computed tomography (CT) imaging. In cases of doubt, fine-needle aspiration for culture may be performed. 1 3

In the present report, we present the case of an AP patient whose clinical course was complicated with acute biliary peritonitis. In general, there are literature reports of biliary peritonitis due to bile duct rupture caused by iatrogenic injuries onto the extra hepatic bile duct system after surgery, endoscopic or percutaneous interventions in the biliary tree, or even abdominal trauma. 4

Spontaneous bile duct rupture is a rare condition in adults with only approximately 70 reported so far. 5 On the other hand, it is more frequent in pediatric population due to the possible presence of congenital abnormalities of the pancreatobiliary ductal system. 6 Increased ductal pressure caused by obstructions or even erosion of the bile duct wall caused by gallstones or tumors and finally ischemic necrosis of the bile duct wall may lead all to bile duct rupture and subsequent biliary peritonitis. 7 There are even fewer reports in the literature of biliary peritonitis complicating the clinical course of AP or chronic pancreatitis. 8 9 A bile duct system rupture due to the inflammatory processes associated with the AP was the identified cause of biliary peritonitis in these cases. However, a rather unusual and unexpected cause of biliary peritonitis was the culprit in the present report.

## Presentation of the Case

A 41-year-old Caucasian female with a known previous history of asymptomatic cholelithiasis presented to the emergency department with low-grade fever and acute right upper quadrant pain, radiating at the back. Several episodes of bile-stained vomiting and diarrheas supplemented the patient's clinical course. The clinical examination revealed mild tenderness in the upper abdomen without signs of peritoneal irritation. Blood tests results showed a total leukocyte count of 13.000 cells/mm3 and C-reactive protein levels of 29mg/mL. Biochemical evidences of cholestasis were noted with direct bilirubin serum levels of 2.8mg/dL. Serum amylase levels were 4000 IU/DL, confirming the diagnosis of AP. An abdominal ultrasound was performed that confirmed the presence of cholelithiasis and a common bile duct (CBD) dilation (13mm in diameter).

**Fig. 1:**
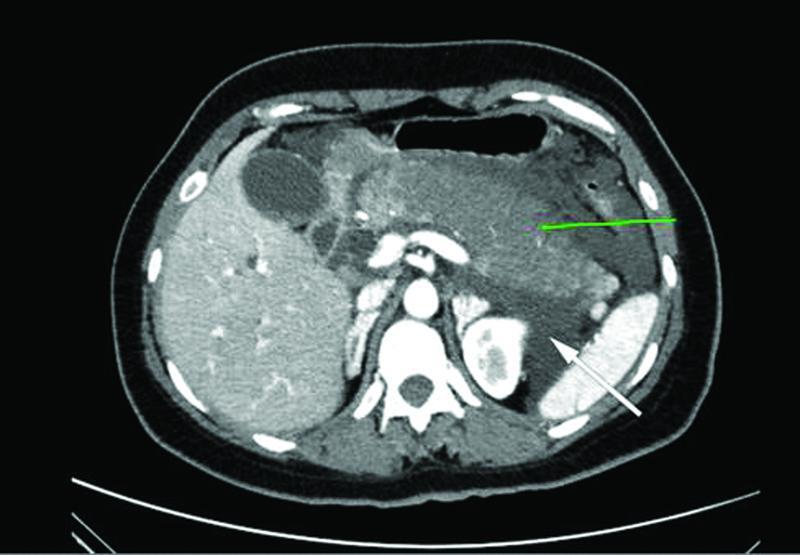
Computed tomography scan image showing acute necrotizing pancreatitis involving the body and tail of the pancreas (green arrow), with acute peripancreatic fluid collections (white arrow).

**Fig. 2:**
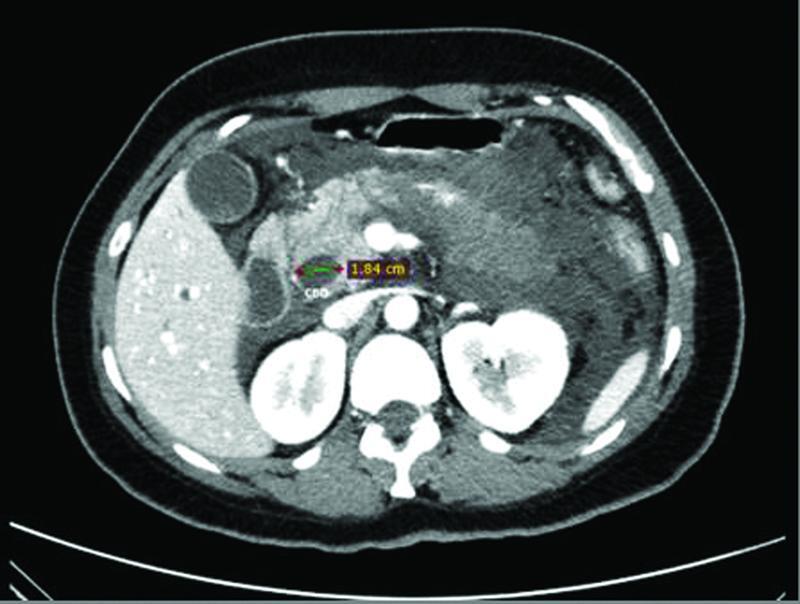
Computed tomography scan image showing the dilated common bile duct (common bile duct diameter: 1.84cm).

The patient remained fastened with intravenous fluids and adequate analgesia and was started on broad-spectrum antibiotics. Three days after the admission and while the patient's symptoms did not effectively alleviate, a contrast enhanced CT scan abdomen was decided revealing evidence of necrotizing AP involving mainly the body and tail of the pancreas with excessive peripancreatic fluid collections ( Fig. 1 ). Cholelithiasis as well as dilation of the CBD (18mm in diameter) was also noted ( Fig. 2 ).

**Fig. 3:**
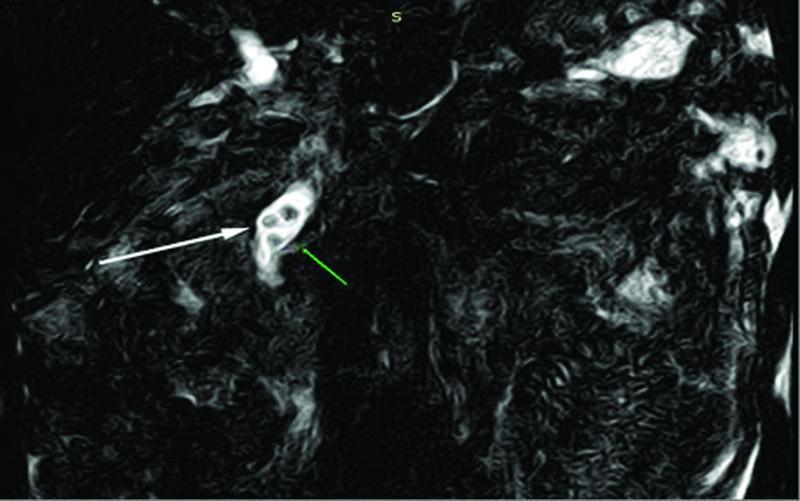
Magnetic resonance cholangiopancreatography image showing the dilated common bile duct (white arrow) with several filling defects consistent with gallstones. The main pancreatic duct (green arrow) is depicted as well.

**Fig. 4:**
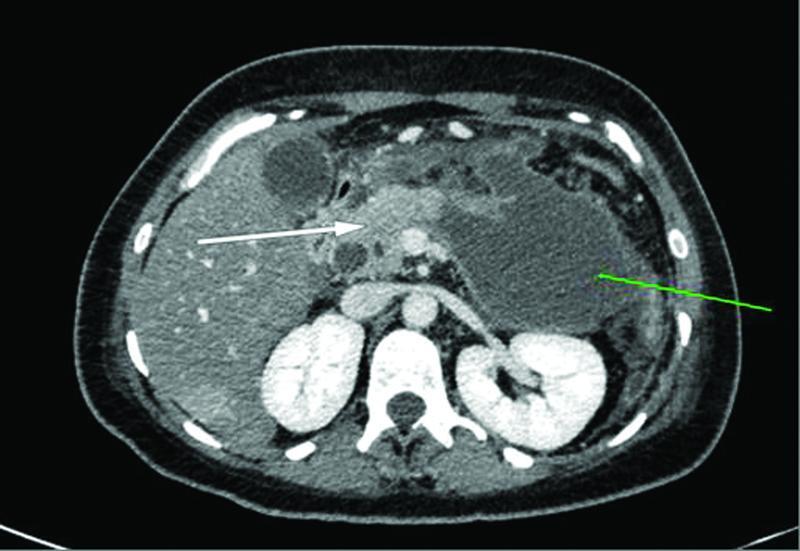
Computed tomography image showing the acute necrotic collection (green arrow) along with diffuse edema of the pancreatic parenchyma (white arrow).

A magnetic resonance cholangiopancreatography (MRCP) was then performed to more accurately assess the biliary tree. The CBD was found dilated with several filling defects consistent with gallstones ( Fig. 3 ). On the absence of evidence of acute cholangitis and because the patient was clinically improving on conservative treatment, the decision to defer the CBD clearance via endoscopic retrograde cholangiopancreatography (ERCP), until the AP episode resolved, was made. However, 18 days after the admission and 2 days after successfully recommencing oral feeding, signs of sepsis, that is, spikes of fever (up to 39.5°C) and increased laboratory markers of inflammation (white blood cell count: 23.000 cells/mm3) combined with signs of peritoneal irritation on clinical examination became evident. A CT scan abdomen was decided revealing an ANC in the anatomic area of the body and tail of the pancreas along with a notable free intraperitoneal fluid collection ( Fig. 4 ).

A decision for an urgent surgical intervention was made. Access to the abdominal cavity was gained through a midline incision. Upon laparotomy, the peritoneal cavity was found filled with several liters of bile-stained fluid consistent with biliary peritonitis. An exploration toward the identification of possible ruptured gallbladder or a ruptured CBD as the cause of the biliary peritonitis proved negative. After properly entering the lesser sac, a bulging, encapsulated, though partially ruptured, necrotic collection of the body of pancreas was identified. Surprisingly, the etiology of biliary peritonitis, that is, the source of bile was the partially ruptured, ill-defined wall of the AP necrosis. Copious irrigation with several liters of normal saline of the pancreatic necrotic collection was then conducted and an external tube drain was left in place.

A decision to decompress the extra hepatic biliary tree was then made. Thus, a cholecystectomy was performed followed by CBD exploration. More specifically, after a straightforward cholecystectomy, using a pointed scalpel and a Pott's right-angled scissors, a 2cm longitudinal incision onto the CBD was performed. With fingers placed behind the duodenum and the head of the pancreas, the CBD was milked retrieving several stones as they emerged from the site of choledochotomy. Using a retrieval basket, followed by repeated bile duct flushing, more stones were retrieved. To ensure the proper CBD clearance, a 5 Fr Fogarty catheter was additionally used. However, even after several attempts we did not manage to pass the Fogarty catheter through the ampulla and into the duodenum. Having in mind the quite possible scenario of residual stones in the most distal part of the CBD, the choledochotomy was closed with interrupted absorbable sutures over a Latex rubber 14-Fr T-tube. An additional drain was left in place.

**Fig. 5:**
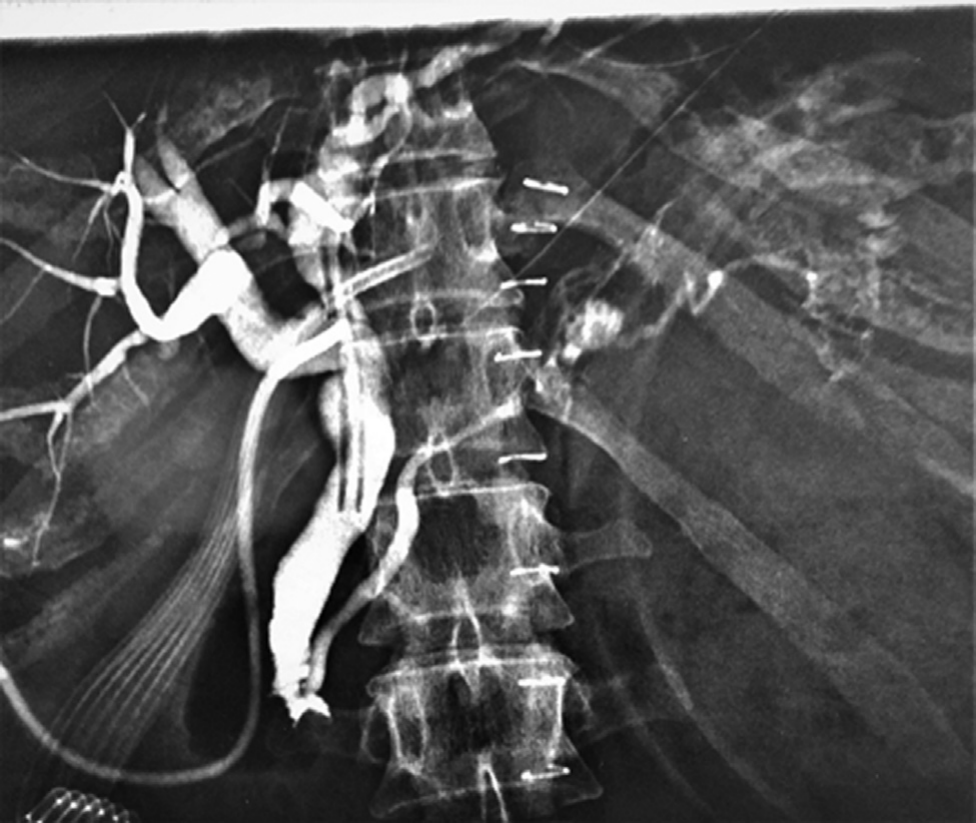
Cholangiogram through the T tube showing the out of the ordinary source of bile into the walled off necrosis.

The postoperative period was relatively uneventful. Clinical improvement was rapid. The left-sided drain output, however, was 1000mL/24hours of bile-stained fluid with sky high concentrations of amylase (58.000 U/L) and bilirubin (42mg/dL). A cholangiogram through the T tube was performed on the 5th postoperative day which confirmed what was highly suspected during laparotomy, that is, the unusual cause of biliary peritonitis in this patient ( Fig. 5 ). Bile due to obstruction at the level of the ampulla regressed through the common channel into the pancreatic duct and finally into the pancreatic necrotic collection.

**Fig. 6:**
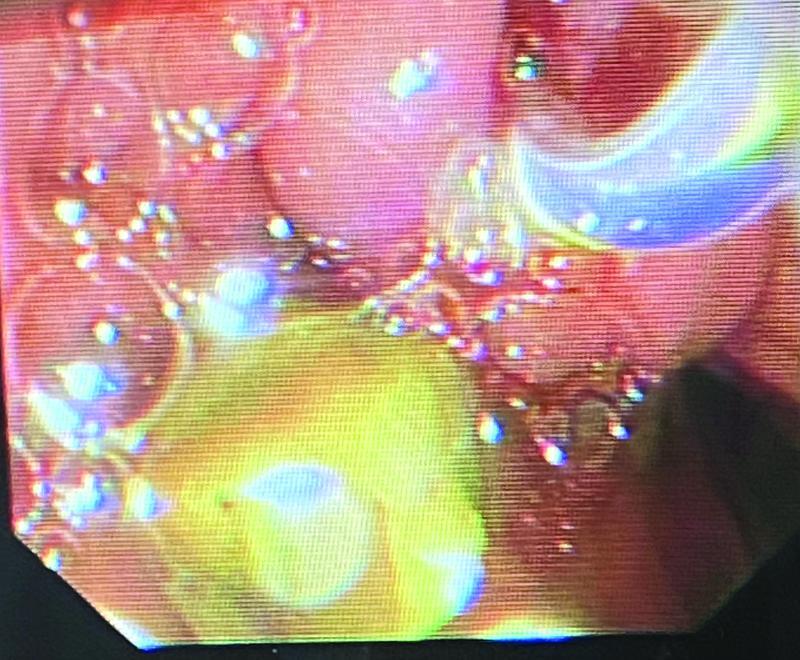
Endoscopic image of the ampulla during endoscopic retrograde cholangiopancreatography.

**Fig. 7:**
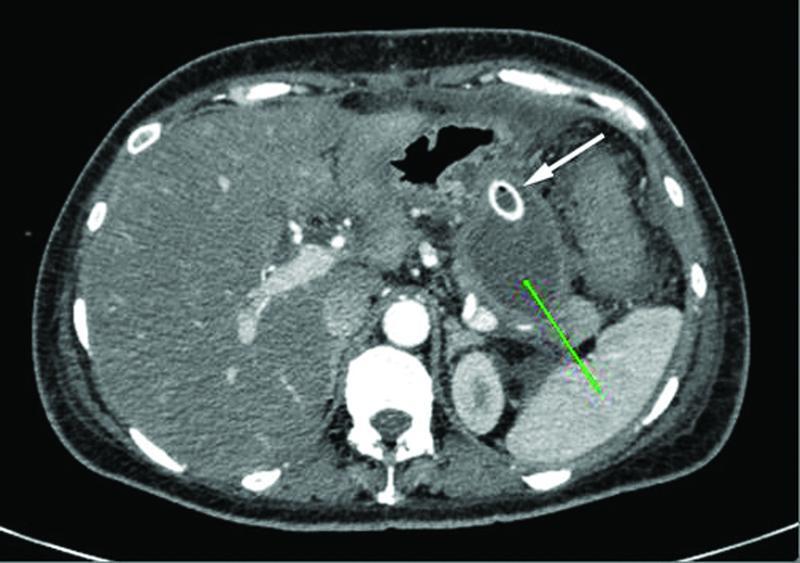
Computed tomography image of the walled off necrosis (green arrow) with the drainage tube (white arrow).

An ERCP was then performed with the intention to remove the residual gallstones and relief the obstruction at the level of the ampulla. Despite fears for technical difficulties during ERCP, the procedure was uneventful and straightforward. During ERCP, after the successful catheterization of the CBD, sphincterotomy was performed and the residual gallstones, causing the obstruction, were effortlessly removed ( Fig. 6 ). An imaging reassessment with CT scan of the abdominal collections revealed markedly decreased dimensions of the pancreatic necrotic collection ( Fig. 7 ). The right-sided drain was removed on the 6th postoperative day, while a gradually decreasing left drain output was observed soon after the successful ERCP. We were able to remove the second drain, when it dried up, on the 14th postoperative day. The patient was discharged on day 16th postoperative day.

## Discussion

The management of patients with AP requires a multimodality approach. Key questions represent the timing and the type of the intervention. The patient with the septic profile where there are obvious signs of infected necrosis is candidate for some kind of intervention. Only, a small minority of these patients can recover with antibiotics alone. 10 The step-up approach strategy, supported by recent literature reports, has gain popularity during the recent years. 11 Escalating interventions from percutaneous or endoscopic drainage to open laparotomy, aiming in reducing morbidity and mortality, is the theory behind this concept. 12 13

Most of the patients without a documented infected necrosis can be ultimately managed conservatively. According to the 2019 WSES guidelines for the management of severe AP, the following are indications for surgical intervention: as the last part of the step-up approach strategy, abdominal compartment syndrome, acute ongoing bleeding not amenable to endovascular treatment, bowel ischemia, acute necrotizing cholecystitis, and development of a bowel fistula. 14

Although, these indications for surgical intervention during the clinical course of AP are well-established and mainly anticipated, we came across a rather unusual case scenario, that is, AP complicated by biliary peritonitis. Despite the fact that there are reports highlighting the possibility of spontaneous CBD rupture on the background of AP, the etiology of biliary peritonitis in the present report was quite different.

A female patient with biliary AP and MRCP documented choledocholithiasis was treated conservatively. On the absence of cholangitis, the initial plan was to perform an ERCP followed by laparoscopic cholecystectomy electively after the episode of AP resolved. However, the patient's clinical condition dictated a more-prompt surgical intervention. As soon as signs of sepsis and peritoneal irritation were established, a decision for an urgent laparotomy was made. The intraoperative findings were consistent with biliary peritonitis and a ruptured pancreatic necrotic collection was the source of bile. A decision to gain access on the extrahepatic biliary tree in the form of CBD exploration after the removal of the gallbladder was made during laparotomy.

After the CBD exploration, there was a high suspicion of residual choledocholithiasis but as there was no option for an intraoperative cholangiogram, the CBD assess incision was closed over a T tube. Postoperatively, the output of the drain placed within the pancreatic necrotic collection was markedly increased and consistent with a pancreatic/biliary fistula, that is, high amylase and bilirubin concentrations. It was the cholangiogram performed through the T tube the crucial test to document what was highly suspected as the cause of the bile leak through the ruptured pancreatic necrotic collection. A gallstone obstructing the CBD, at the level of the ampulla, was causing bile to reflux into the main pancreatic duct and subsequently into the ruptured necrotic collection.

In conclusion, AP can cause a wide variety of local complications sometimes pretty unusual. Dealing with the unexpected is a constant challenge for the surgical team dealing with AP patients. Although deferring surgical intervention during the course of AP as much as possible is the ideal strategy, this is not always possible. Deciding the treatment strategy based on the patients' clinical condition represents the most appropriate approach. In our case, the utilization of the available interventional and surgical treatment options, under the proper indications, resulted in the successful outcome of a rather challenging AP case.
